# Control of mucosal virus infection by influenza nucleoprotein-specific CD8^+ ^cytotoxic T lymphocytes

**DOI:** 10.1186/1465-9921-8-44

**Published:** 2007-06-27

**Authors:** Innocent N Mbawuike, Yongxin Zhang, Robert B Couch

**Affiliations:** 1 Viral Respiratory Pathogens Research Unit, Departments of Molecular Virology and Microbiology, Baylor College of Medicine, Houston, Texas, 77030, USA

## Abstract

**Background:**

MHC class I-restricted CD8^+ ^cytotoxic T lymphocytes (CTL) are thought to play a major role in clearing virus and promoting recovery from influenza infection and disease. This has been demonstrated for clearance of influenza virus from the lungs of infected mice. However, human influenza infection is primarily a respiratory mucosal infection involving the nasopharynx and tracheobronchial tree. The role of CD8^+ ^CTL directed toward the influenza nucleoprotein (NP) in defense against influenza virus infection at the respiratory mucosa was evaluated in two separate adoptive transfer experiments.

**Methods:**

Influenza nucleoprotein (NP)-specific CD8^+ ^CTL were generated from splenocytes obtained from Balb/c mice previously primed with influenza A/Taiwan/1/86 (H1N1) infection or with influenza A/PR/8/34 (H1N1)-derived NP plasmid DNA vaccine followed by infection with A/Hong Kong/68 (H3N2) virus. After *in vitro *expansion by exposure to an influenza NP-vaccinia recombinant, highly purified CD8^+ ^T cells exhibited significant lysis *in vitro *of P815 target cells infected with A/Hong Kong/68 (H3N2) virus while the CD8^- ^fraction (CD4^+ ^T cells, B cells and macrophages) had no CTL activity. Purified CD8^+ ^and CD8^- ^T cells (1 × 10^7^) were injected intravenously or interperitoneally into naive mice four hours prior to intranasal challenge with A/HK/68 (H3N2) virus.

**Results:**

The adoptively transferred NP-vaccinia-induced CD8^+ ^T cells caused significant reduction of virus titers in both the lungs and nasal passages when compared to CD8^- ^cells. Neither CD8^+ ^nor CD8^- ^T cells from cultures stimulated with HIV gp120-vaccinia recombinant reduced virus titers.

**Conclusion:**

The present data demonstrate that influenza NP-specific CD8^+ ^CTL can play a direct role in clearance of influenza virus from the upper respiratory mucosal surfaces.

## Background

Studies in mice have shown definitively that MHC class I-restricted CD8^+ ^CTL can promote recovery from pneumonia caused by an influenza virus infection [1,2]. Proof was provided using adoptive transfer of CD8^+ ^T cells and clones [3,4,1], *in vivo *depletion of CD8^+ ^T cells using monoclonal antibodies from mice previously infected with influenza virus [5,6] and transgenic CD8^+ ^T cell knockout mice [7]. CD8^+ ^CTL mediated clearance of lung virus infection, reduced pneumonia severity and prevented mortality from the influenza virus infection.

Despite availability of data for mice, a beneficial role for CTL activity in promoting clearance of influenza virus infection in human influenza cannot be assumed because influenza in humans is primarily an infection of the mucosal surface of the respiratory tract; pneumonia can occur but this is uncommon [8]. Thus, for CTLs to be of value for human influenza, they must mediate control of virus infection of respiratory tract epithelial cells. In this regard, it has been suggested that CTLs in the circulation or submucosal sites cannot cross the basement membrane of the mucosal surface; this should be necessary to exert an effect [9]. Studies by McMichael and coworkers demonstrated a correlation between human HLA-restricted CTL activity and reduced viral shedding from the nasopharynx of humans; however a contribution from antibody could not be excluded [10].

CD8^+ ^CTL for influenza virus infection are directed predominantly toward antigens on the nucleoprotein (NP) [11-14]. Antibody to NP has clearly been shown to have no role in prevention or recovery from infection [15,16] so a beneficial immune response directed specifically toward the NP would be a cell mediated immune response; antibodies that could contribute to the control of influenza are directed toward the hemagglutinin and neuraminidase surface proteins [15] or the M2 protein [17]. The objective of the present study was to define the role of CD8^+ ^CTL directed toward the NP protein in defense against influenza virus infection of the respiratory mucosa.

## Methods

### Mice

Eight- to twelve- week old Balb/c (H-2^d^) mice were purchased from Charles River Laboratories under a contractual arrangement with the National Institute on Aging and were housed in specific pathogen-free-certified rooms in cages covered with barrier filters and with sentinel cages to monitor infections.

### Influenza viruses

Virulent challenge pools of mouse-adapted influenza viruses A/Hong Kong/1/68 (A/H3N2) and A/Taiwan/1/86 (A/H1N1) viruses were prepared by serially passaging each virus in mice as described previously [18-20]. The NP of A/HK/68 and A/Taiwan/1/86 viruses are antigenically similar to each other and to A/PR/8/34 (H1N1) virus NP while the HA and NA surface glycoproteins of A/HK/68 and A/Taiwan/86 are antigenically distinct. A mouse fifty percent lethal dose (MID_50_) following administration by small particle aerosol (SPA) was determined for each virus as described previously [18-20]. A mouse fifty percent infectious dose (MID_50_) was determined following intranasal (i.n.) administration of a small volume (10 μl) of virus suspension. Viruses for use in CTL assays were prepared as described previously by us [18-20].

### DNA vaccine

An influenza NP plasmid DNA vaccine was obtained from Drs. Margaret Liu and Donna Montgomery of Merck Research Laboratories, West Pont, PA [21,22]. The expression vector system (VIJ) consists of a pUC19 backbone with an IE1 enhancer promoter intron A of hCMV (CMVintA) and a bovine growth hormone (BGH) polyadenylation (poly A) signal sequence for driving the expression of the reporter gene chloramphenicol acetyltransferase (CAT) or the influenza protein [21]. The NP gene from influenza A/PR/8/34 was cloned into the *Bgl*II and *Sal*I sites. Plasmid DNA used for vaccination was purified from *E. coli *(DH5a) containing VIJ-NP by a modified alkaline lysis procedure using QIAGEN (Chatsworth, CA) Giga Plasmid Purification kits. The DNA was banded twice on CsCl_2 _gradients.

### Vaccinia viral gene recombinants

Thymidine kinase-negative (TK-) recombinant vaccinia virus expressing influenza A/PR/8/34 NP gene (NP-Vac) was provided by Dr. Bernard Moss, NIH, Bethesda, MD [23]. Vaccinia recombinant expressing HIV gp120 (Vac-gp120) [24,25] was obtained from the AIDS Research and Reference Reagents Program, Division of AIDS, NIAID, NIH.

### Immunizations

Mice were infected with live virus by administering 0.05 LD_50 _A/Taiwan/86 by small particle aerosolization [19,18,20]. For DNA vaccination, mice were anesthetized by subcutaneous injection of 25 μl of ketamine-xylazine-acepromazine cocktail (37.5/1.9/0.37 mg/ml). The legs of the mice were then flooded with 70% ethanol and NP DNA (200 μg) was injected into each anterior tibial muscle 3 times, 2 weeks apart. Three weeks after the last dose, DNA-immunized mice were challenged with 1 LD_50 _of A/HK/68 (H3N2) virus by small particle aerosol. Sixty to eighty percent of the mice survived.

### Generation of secondary CTL activity and chromium release assay for CTL

Spleen cells were obtained from mice immunized with A/Taiwan/86 and from DNA-immunized mice surviving A/HK/68 virus challenge (after 3–4 months). Influenza virus-specific and NP-specific CTL were generated as previously described [20,26]. Briefly, stimulator cells were prepared by infecting spleen cells with A/HK/68 (H3N2) virus and washing. In addition, cells were infected with 1 pfu of Vac-NP or Vac-gp120 for 2 hours and irradiated using a cesium source (2500 rads). The stimulators were then co-cultured with responder cells at a 1:10 ratio for 5 days. CTL effectors were harvested, depleted of dead cells using Ficoll Hypaque gradient centrifugation. Tests for CTL activity against A/HK/68-infected P815 (H-2^d) ^target cells was assessed in a 4-hour ^51^Cr release assay [20,26].

### Purification of CD8^+ ^T cells

CD8^+ ^T cells were purified by negative selection using the magnetic affinity cell sorting MACS method [20,26,27]. Alternatively cells were purified by positive selection using AutoMacs mini cell sorter (Miltenyi Biotec, Auburn, CA). Briefly, effector cells (10^7^) were incubated with 20 μl of magnetic CD8a (Ly-2) MicroBeads™ (Miltenyi Biotec; MicroBeads conjugated to rat monoclonal anti-mouse CD8a Ly-2 antibody) for 30 minutes at 4°C and washed. After passing through a column placed in the magnetic field of an AutoMacs, purified CD8^+ ^T cells and CD8^- ^cells (CD4^+ ^T cells, B cells and macrophages) were eluted. The frequency of CD8^+ ^and CD4^+ ^cells in each fraction was determined using PE-conjugated rat anti-mouse CD8a (Ly-2) (clone 53-6.7) and FITC-conjugated Rat Anti-Mouse CD4 (L3T4), IgG2b, clone GK1.5, BD Biosciences) by dual color flow cytometry (Beckman Coulter, Miami, FL). The AutoMacs positive selection is more sensitive than the MACS technique, but the purity of target cells are similar.

### Adoptive transfer of T lymphocytes and virus challenge

Purified CD8^+ ^T cells and CD8- cells were suspended in PBS at 1 × 10^7 ^cells/ml. Using a 27 gauge needle, naïve mice were injected in the tail vein or intraperitoneally with a 0.1 ml volume to deliver 1 × 10^7 ^cells. Four hours later, they were challenged by intranasal inoculation with 10 μl containing 100–200 MID_50 _of A/HK/68 (H3N2) virus. Lung and nasopharyngeal virus titers were assessed 8 days later.

### Quantitation of lung and nasopharyngeal virus

Lungs from infected mice were obtained aseptically at different times following virus challenge and homogenized in vials containing 1 μm glass beads using a Mini-bead beater (Biospec Products, Bartlesville, OK) as previously described [26-28]. For nose washes, jaws of the dead animals were disarticulated and then removed. One ml of 2% FCS-MEM was pushed through each nostril and the effluent collected from the posterior opening of the pallet. Nasal turbinates were also isolated and homogenized as for lungs. Lung, nasal wash and turbinate specimens were titrated for influenza virus in microplates of MDCK tissue cultures [29,28,26].

### Data analysis

The Mann Whitney nonparametric analysis was used to compare geometric mean titers in different groups. Lung and nasal turbinate homogenates and nose wash samples with undetectable virus titers (< 1 TCID50) were assigned a value of 0.7 TCID_10 _for statistical evaluation. These tests were performed using STATVIEW Software (SAS Institute, Inc, Cary, NC). A difference between comparison groups of p < 0.05 level was considered significant.

## Results

### Characterization of purified CD8^+ ^T cells

**Figure 1:**
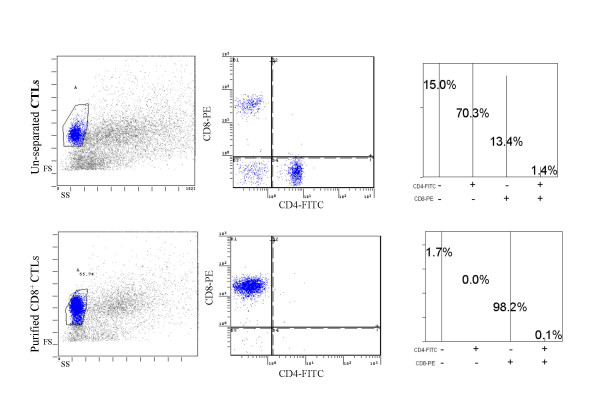
Characteristics of purified T cells. The frequency of CD8^+ ^and CD4^+ ^T cells before (top) and after (bottom) separation using AutoMacs are presented. Effector CTLs were labeled with magnetic CD8a (Lyt-2) MicroBeads and CD8^+ ^and CD8^- ^cells collected by passage through a magnetized column. The cells were stained with CD8-PE and CD4-FITC conjugated monoclonal antibodies, respectively. Forward (FS) and side scatter (SC) plots of total lymphocytes (left), two-parameter histogram of gated CD8-PE^+^/CD4-FITC^+ ^cells (middle) and prism display of cell frequencies are shown for un-separated CTL (top) and purified CD8^+ ^CTLs (bottom) are shown a representative sample.

CTL effector cells were depleted of dead cells and then labeled with anti-CD8 MicroBeads and isolated using the AutoMacs cell sorter (Miltenyi Biotec); cell recovery is usually low (< 50%). The purity of CD8^+ ^T cells was determined using dual color (anti-CD4-FITC and anti-CD8-PE) flow cytometry; Figure 1 shows the results of a typical phenotypic analysis. Prior to separation, 13.4% of the effector cells were CD8^+ ^while 70.3% were CD4^+^; after purification, greater than 98% of the cells were CD8^+ ^T cells while CD4^+ ^cells were undetectable.

Table 1 shows the frequency of CD8^+ ^and CD4^+ ^T cells and the corresponding influenza-specific CTL activity in the CD8^- ^and CD8^+ ^fractions after positive isolation of CD8^+ ^T cells in a typical experiment. Following 5 days of *in vitro *stimulation with vaccinia-NP, the CD8^+ ^cells exhibited significant lysis of P815 (H-2d) target cells infected with influenza A/HK/68 (H3N2) and vaccinia-NP but failed to lyse vaccinia-gp120- and B/Panama influenza-infected target cells. While similar cell frequencies were used for vaccinia-gp120-stimulated cells, the CD8^+ ^T cells did not display significant lysis of influenza A/HK/68 virus, vaccinia-NP- infected or vaccinia-gp120-infected target cells. The CD8^- ^fractions also failed to exhibit appreciable lysis of any of the target cells. Influenza A/HK/68-infected EL-4 (H-2^b^) target cells were not lysed by NP-specific CD8^+ ^T cells (data not shown). These results confirm influenza A virus specificity and MHC class I restriction of the cells used for adoptive immunizations.

**Table 1:** Specificity of purified CD8^+ ^CTL ^a^

				**% specific lysis ^b^**			
**Stimulation**	**Cell Fraction**	**% CD8^+ ^cells**	**% CD4^+ ^cells**	**A/HK/68**	**Vac-NP**	**Vac-gp120**	**B/Panama**

Vac-NP	CD8^+^	97.3	1.1	22.4	44.3	1.3	0.0
	CD8^-^	0.6	75.5	NT	1.1	NT	2.7
Vac-gp120	CD8^+^	90.1	0.0	0.34	0.0	9.02	NT
	CD8^-^	0.0	74.2	NT	3.3	NT	NT

^a ^Splenocytes from Balb/c mice primed with influenza A/Taiwan (H1N1) virus were stimulated with 2 pfu/cell of recombinant Vac-NP or vaccinia-gp120 for 5 days. CD8^+ ^T cells were obtained by positive selection using AutoMacs instrument. The percentage of CD8^+ ^and CD4^+ ^T cells determined by dual color flow cytometry and % lysis in purified CD8^+ ^and CD8^- ^fractions are presented. ^b ^Lysis of P815 (H-2^d^) target cells with the indicated infection was determined in the 4-hour chromium release assay. Percent specific lysis at 40:1 E:T ratio is shown. NT = not tested.

### Protective effects of NP-specific CD8^+ ^

#### i) T cells from influenza infected mice

**Figure 2:**
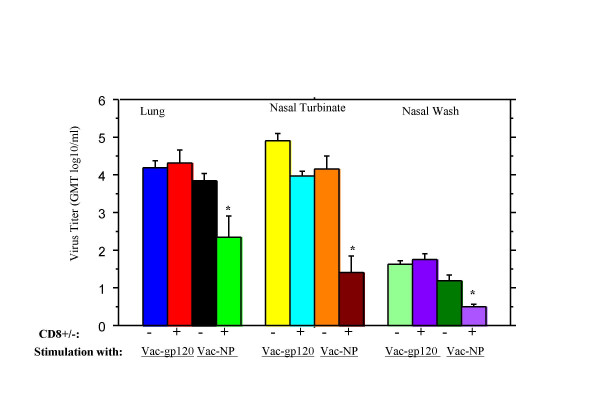
Protective effects of CD8^+ ^CTLs from influenza-infected mice. Splenic lymphocytes from Balb/c mice surviving A/Taiwan (H1N1) infection were stimulated with vaccinia-NP and vaccinia-gp120, respectively, for 5 days. CD8^+ ^and CD8^- ^cells (CD4^+ ^T cells, B cells and macrophages) were obtained using AutoMacs. 1 × 10^7 ^cells were injected into the tail vein of the mice (6 per group). Four hours later, they were challenged by intranasal inoculation of 200 MID50 of influenza A/HK68 virus. Virus in the lung, nasal turbinate and nasal wash was measured 8 days later. * P < 0.005. Values are GMT log_10_/ml of lung or nasal turbinate homogenate ± SEM.

Previous studies have shown that influenza-induced CD8^+ ^T cells reduced pulmonary influenza infection following adoptive transfer. To confirm this and to evaluate the protective effect of CD8^+ ^T cells at the upper respiratory mucosal site in the same mice, Balb/c mice were infected with 0.05LD_50 _of A/Taiwan/1/86 (H1N1) virus. Splenocytes were harvested three to four months later and stimulated *in vitro *for five days with vaccinia-NP or vaccinia-gp120. After stimulation, ten million purified CD8^+ ^T cells or CD8^- ^cells isolated by AutoMacs were injected via the tail vein into naïve Balb/c mice. Four hours later, they were challenged by intranasal inoculation of 10 μl containing 200 MID_50 _of A/HK/68 virus (without anesthesia). Virus titers in the nasal passages and lungs were assessed on day 8. This time period permits assessment of virus clearance because CD8^+ ^T cells do not prevent virus infection and differences may not be detected early after infection [26]. In normal mice, influenza virus replication peaks at three for four days and cleared by approximately twelve days [26,18,20]. Figure 2 shows that transfer of CD8^+ ^T cells stimulated *in vitro *with vaccinia-NP induced a significant reduction of virus replication in both lung, nasal turbinate and nasal passages (p < 0.001–0001) when compared to CD8^- ^cells from vaccinia-NP stimulated cultures. CD8^+ ^T cells from vaccinia-gp120 stimulated cultures did not reduce virus titers. Thus, influenza NP-specific CD8^+ ^CTL from influenza infected mice reduced lung and nasal virus replication following challenge with influenza A virus of a different subtype.

#### ii) T cells from plasmid NP DNA vaccinated mice

**Figure 3:**
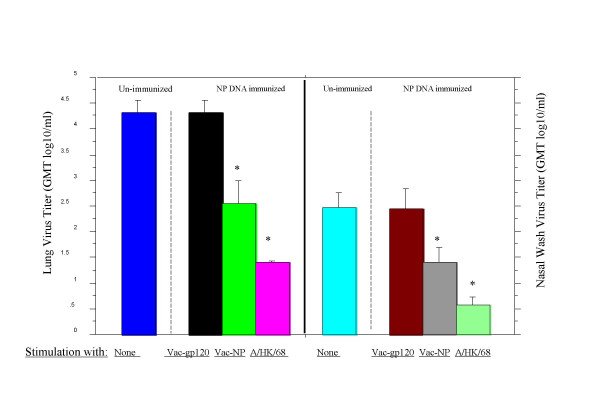
Protective effects of CD8^+ ^T cells from plasmid NP DNA vaccinated mice. Balb/c mice were immunized intramuscularly with 200 μg of influenza A/PR/8/34 (H1N1) NP DNA vaccine, 2–3 weeks apart, three times, and then challenged with 1LD_50 _of influenza A/HK/68 (H3N2) virus. Three months later, spleen cells from survivors were stimulated with Vac-NP, Vac-gp120 or A/HK/68 virus for 5 days. Unstimulated cells from naïve mice served as negative controls. CD8^+ ^T cells were enriched using MACS and injected interperitoneally into naïve mice (1 × 10^7 ^cells per mouse). Four hours later, they were challenged with 100 MID_50 _of A/HK/68 virus (in 10 μl intranasally). Lung and nasal wash virus titers were determined after 8 days. * p < 0.05 to 0.001, Vac-NP versus Vac-gp120. Values are GMT log_10_/ml of lung homogenate or nasal wash ± SEM. (4–6 mice per group).

NP DNA immunization of mice prevented mortality after challenge with virulent influenza viruses (data not shown). Thus, NP DNA immunized mice that survived influenza A virus challenge should contain high numbers of NP-specific CD8^+ ^T cells. To further determine whether NP-specific CD8^+ ^CTL play a role in the control of influenza virus in the upper respiratory mucosa, NP-specific CD8^+ ^CTL from the NP DNA immunized mice that survived an A/Hong Kong/68 virus challenge were expanded *in vitro *three months later and used for adoptive transfer studies. Splenocytes were harvested and stimulated with vaccinia recombinants encoding influenza NP, HIV gp120 (negative control) or with influenza A/HK/68 (H3N2) virus for 5 days; a negative control consisted of un-stimulated splenocytes from un-immunized mice. Induced effector cells were enriched for CD8^+ ^CTL by MACS or AutoMacs. Ten million CD8^+ ^T cells were injected intraperitoneally into naive Balb/c mice; 4 hours later they were challenged intranasally with a small volume of saline (10 μl) containing 100 MID_50 _of an influenza A/HK/68 (H3N2) virus under light Metofane anesthesia. Virus quantities in nasal passages and lungs were assessed 8 days later. [The small inoculum IN challenge was used to ensure that virus was deposited primarily in the nose [30].] Adoptive transfer of CD8^+ ^CTL from cultures stimulated with Vac-NP or live influenza A/HK/68 resulted in significant reduction in lung (Figure 3, left panel) and nasal wash (Figure 3, right panel) virus titers when compared to mice given unstimulated unimmunized or Vac-gp120-stimulated CD8^+ ^T cells. These results showed that CD8^+ ^T cells generated from the NP DNA-vaccinated mice that survived influenza A virus challenge could reduce virus shedding at the upper respiratory mucosal surface.

## Discussion

The present study was initiated to determine whether CD8^+ ^T cells alone can control an influenza virus infection in the epithelial cells lining the respiratory tract because human influenza virus infection is primarily a mucosal infection with a significant involvement of the upper respiratory tract. In previous reported studies, adoptive transfer of unfractionated influenza immune T cells or cloned influenza-specific CD8^+ ^T cells [4,31,1], resulted in significant clearance of influenza virus from the lungs and protection against pneumonia-induced death among the recipient mice. Although, depletion of T cell subsets *in vivo *suggested that CD8^+ ^T cells were required for clearance of the lung influenza virus infection [5,6], CD8^+ ^deficient mice results were inconsistent [32,33,7]. This was due possibly to unpredictable compensatory mechanisms in these gene knock out animals [34-36]. Although above studies demonstrated or suggested a role for CD8^+ ^cells in the mouse pneumonia model of influenza, they did not evaluate whether they functioned in clearance of influenza virus from the respiratory mucosa.

In the present study, highly purified NP-specific CD8^+ ^CTL, were shown to mediate clearance of influenza infection from both the lung and nasal mucosa of influenza-infected mice. The NP-specific CD8^+ ^CTL were generated from mice immunized with plasmid NP DNA vaccine that survived influenza A virus challenge and from mice immunized with live influenza virus infection by stimulation in vitro with a vaccinia-NP vector. Control CD8^+ ^T cells stimulated with vaccinia-gp120 did not kill influenza virus-infected target cells in vitro and did not mediate clearance of virus in vivo. In addition CD8^- ^cells (including CD4^+ ^T cells, B cells and macrophages) did not mediate clearance of virus. Thus, the present adoptive transfer experiments demonstrate that NP-induced CD8^+ ^CTL can promote clearance of influenza virus infection in epithelial cells lining the respiratory tract. Moreover, we found no evidence for a direct role for CD4^+ ^T cells in viral clearance although they may be important in supporting the development of CD8^+ ^effector T cells [37,38,6]. Data from some laboratories have indicated that CD4^+ ^T cells are not required either for the induction or function of CD8^+ ^CTL [37] while other studies suggest otherwise [38,6].

Two studies concluded that NP specific T cells do not exhibit antiviral activity *in vivo *[39,32]. In one study, mice immunized with influenza NP-vaccinia and challenged with heterotypic influenza A virus had significantly reduced lung virus titers when the mice were challenged 9 and 30 days following immunization but the authors concluded that NP-induced CTLs did not protect against influenza virus challenge [32]. In the second study, mice were immunized with vaccinia constructs expressing NP_147–155 _epitopic peptide and then challenged 9 days later with a lethal dose of heterotypic influenza A virus [39]. These NP peptide vaccinia constructs failed to reduce lung virus titers and mortality but the CTLs were directed to a single NP epitope and were probably inadequate to protect against a lethal influenza challenge.

The role of contact-mediated T cell cytotoxicity against cytopathic viruses such as influenza remains controversial. Some studies have suggested that cytokines released by T cells and neutralizing antibodies constitute the primary protective mechanisms [9]. In a study by Doherty's group, it was shown that mice primed with influenza A/H1N1 virus clear an A/H3N2 influenza challenge 2–3 days earlier than naïve mice [40]. It was further shown that virus-specific CD8^+ ^T cells produce IFN-γ within six hours while it takes 4–5 days for CD8^+ ^T cells to accumulate in the infected local mucosal epithelium [40]. In those studies, the kinetics of reduction in lung virus titer preceded the accumulation of virus-specific NP_366–474_tetramer^+ ^CD8^+ ^T cells, but reductions partially overlapped peak accumulation of the CD8^+ ^T cells. Other studies using perforin and Fas deficient chimeric mice (P-/-/Fas-/-) showed that CD8^+ ^CTL could clear influenza virus infection via a perforin-dependent pathway and, in the absence of perforin, via virus-infected lung cells expressing Fas [41]. These results are indicative of a contact-dependent mechanism requiring perforin and/or Fas. The results presented here represent a direct demonstration that CD8^+ ^T cells constitute an effective mechanism for clearance of influenza virus infection in the upper respiratory mucosa. Thus, the suggestion that antigen-specific CTLs cannot cross the basement membrane to function effectively on infected epithelial cells does not appear to be true [9].

## Conclusion

These results support an effector role for CD8^+ ^CTL activity in the results of McMichael and coworkers who showed an inverse correlation between influenza virus shedding from the nose and the level of HLA-restricted CD8^+ ^CTL activity in the blood of human volunteers challenged with an influenza virus [10]. In this regard, it is important to emphasize that the function of CD8^+ ^CTL is not to prevent viral infection but rather to mediate clearance of an infection and, thereby, promote recovery from disease and a reduction in disease severity [42]. Currently available data indicate that prevention of influenza infection requires induction of antibody to the surface viral hemagglutinin (HA) or the NA in both serum and respiratory secretions [43]. An obvious implication of the present findings is that NP-specific CD8^+ ^CTL activity can augment protection against influenza induced by antibody and is a desirable immune response for influenza vaccines.

## Competing interests

The author(s) declare that they have no competing interests.

## Authors' contributions

INM planned the experiments, participated in executing most experiments including, vaccination, virus challenge, CTL analysis and manuscript preparation. YZ prepared the DNA vaccine and performed experiments including flow cytometry and CTL analysis. RBC conceived the study, participated in study design, data analysis and writing the manuscript. All authors have read and approved the final version of this manuscript.
